# Effects of complete home-based biofeedback therapy on insomnia disorders in patients with cancer

**DOI:** 10.3389/frsle.2025.1510293

**Published:** 2025-03-24

**Authors:** Hideaki Hasuo, Keita Mori, Hiromichi Matsuoka, Mariko Shimazu, Hisaharu Shizuma, Yukihiro Morita, Hideki Ishikawa

**Affiliations:** ^1^Department of Psychosomatic Medicine, Kansai Medical University, Osaka, Japan; ^2^Clinical Research Support Center, Shizuoka Cancer Center, Shizuoka, Japan; ^3^Department of Psycho-Oncology, National Cancer Center Hospital, Tokyo, Japan; ^4^Department of Molecular-Targeting Prevention, Kyoto Prefectural University of Medicine, Kyoto, Japan

**Keywords:** heart rate variability biofeedback, insomnia disorders, patients with cancer, resonance frequency breathing, home-based therapy, self-efficacy, sleep medication adherence

## Abstract

**Background:**

Professional palliative care for patients with cancer focuses on symptom management.

**Methods:**

This exploratory, randomized controlled study was conducted to compare the effects of complete home-based heart rate variability biofeedback (HRV-BFB) using an estimation formula for resonance frequency in managing insomnia disorders among patients with cancer.

**Results:**

Only 28 patients were included from the initial enrollment of 30 patients from two study sites. Results showed that compared to the control group, the HRV-BFB group improved sleep efficiency from 82.0% (standard deviation: 9.1%) to 87.8% (standard deviation: 7.2%) (*p* < 0.001) and decreased use of sleeping medication from 88.2% (95% confidence interval, 73.6–95.8) to 51.5% (95% confidence interval, 41.8–61.1) (*p* < 0.001).

**Conclusions:**

This suggests that complete home-based HRV-BFB using an estimation formula for resonance frequency may be a promising tool for treating insomnia disorders in patients with cancer, potentially improving sleep efficiency and decreasing reliance on sleep medications.

**Clinical trial registration:**

UMIN 000046884. Registered February 11, 2022. https://center6.umin.ac.jp/cgi-open-bin/ctr/ctr_view.cgi?recptno=R000053496.

## Introduction

Current insomnia treatment is largely reliant on pharmacotherapy, which raises concerns on decreased self-efficacy, adverse effects, and drug dependence (Riemann and Perlis, 2009). Symptom management has gained traction as a form of early palliative care, contributing to the improvement of quality of life and self-efficacy of patients with cancer (Yoong et al., 2013). Heart rate variability biofeedback (HRV-BFB), a form of behavioral therapy and coping skill, is a physiological tool that evaluates HRV through measuring devices. This provides real-time visual feedback on normally imperceptible physiological data, enabling self-regulation of the mind and body. HRV-BFB is well-established in the field of psychiatry (Lehrer et al., 2020) and has been reported to be effective for sleep management (Sakakibara et al., 2013; Hasuo et al., 2020). Notably, to increase HRV in HRV-BFB, resonance-frequency breathing utilizes respiratory sinus arrhythmia, which increases during non-rapid eye movement sleep (Lehrer et al., 2020; Bonnet and Arand, 1997).

Home-based coping is crucial for managing sleep at home, considering the significant physical and temporal burden of hospital visits for patients with cancer. Cognitive behavioral therapy for insomnia is the gold standard treatment for insomnia in cancer patients (Gao et al., 2022). However, it usually requires a visit to a specialized facility, which is time-consuming for patients. On the other hand, HRV-BFB was traditionally limited to specialized facilities, but recent advances in HRV technology have allowed patients to practice it at home. A recent systematic review reported several studies on training HRV-BF at home due to the miniaturization of BF equipment (Lalanza et al., 2023). Meanwhile, a review of patients with cancer included only one study from our group on insomnia (Spada et al., 2022). We reported the utility of a coordinated medical system for insomnia disorders in patients with cancer, which involved resonance frequency identification, in-hospital resonance frequency breathing introduction, and subsequent home practice before bedtime (Hasuo et al., 2023). Resonance frequency breathing in HRV-BFB involves breathing at a specific frequency that maximizes HRV (resonance frequency), which is an indicator of autonomic nervous function (Lehrer et al., 2020). Although resonance frequency is only measurable in specialized medical facilities, we recently developed a formula to estimate the resonance frequency of patients with cancer based on individual characteristics (height and sex) (Hasuo et al., 2024), allowing the practice of home-based HRV-BFB before bedtime. In this study, an equation for estimating resonance frequency was developed using multiple regression analysis of individual characteristics and resonance frequency in healthy volunteers. The adjusted R-squared values were 0.55 for males and 0.47 for females. Furthermore, this estimation equation was shown to be applicable to patients with incurable cancer.

We hypothesized that a complete home-based HRV-BFB using an estimation equation for resonant frequency would be effective for treating insomnia disorder in patients with cancer.

## Materials and methods

Building on our previous work, we aimed to develop and evaluate a complete home-based HRV-BFB using an estimation formula for resonance frequency, assessing its impact on insomnia disorders in patients with cancer compared to conventional care in a randomized, open-label, controlled study. The participants were patients with cancer and insomnia from two hospitals. Key selection criteria included the following: (1) pathologically diagnosed cancer (including cancer survivors with no recurrence) and (2) clinically diagnosed insomnia disorder based on the Fifth Edition of the Diagnostic and Statistical Manual of Mental Disorders. Main exclusion criteria included sleep-wake disorders other than insomnia (e.g., breathing-related sleep disorders, restless legs syndrome). The study informed consent was taken from all the patients.

**Figure 1:**
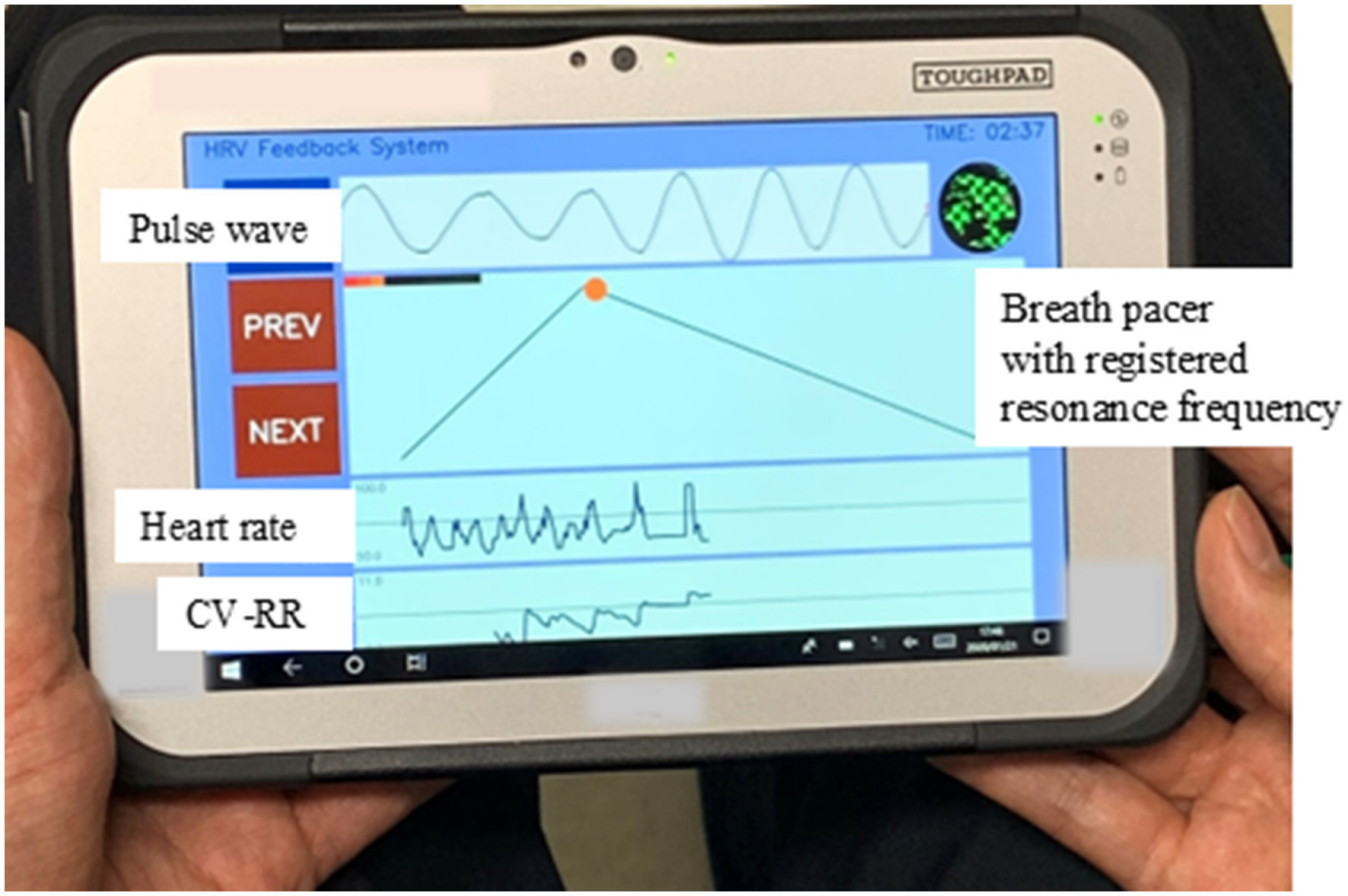
Study flowchart. HRV, heart rate variability; BF, biofeedback, PSQI-J, Japanese version of the Pittsburgh Sleep Quality Index.

Utilizing a computer-generated algorithm, participants were randomly allocated (1:1) to either home-based HRV-BFB with conventional care (HRV-BFB group) or conventional care alone (control group) through central registration by an independent third-party organization (Figure 1).

**Figure 2:**
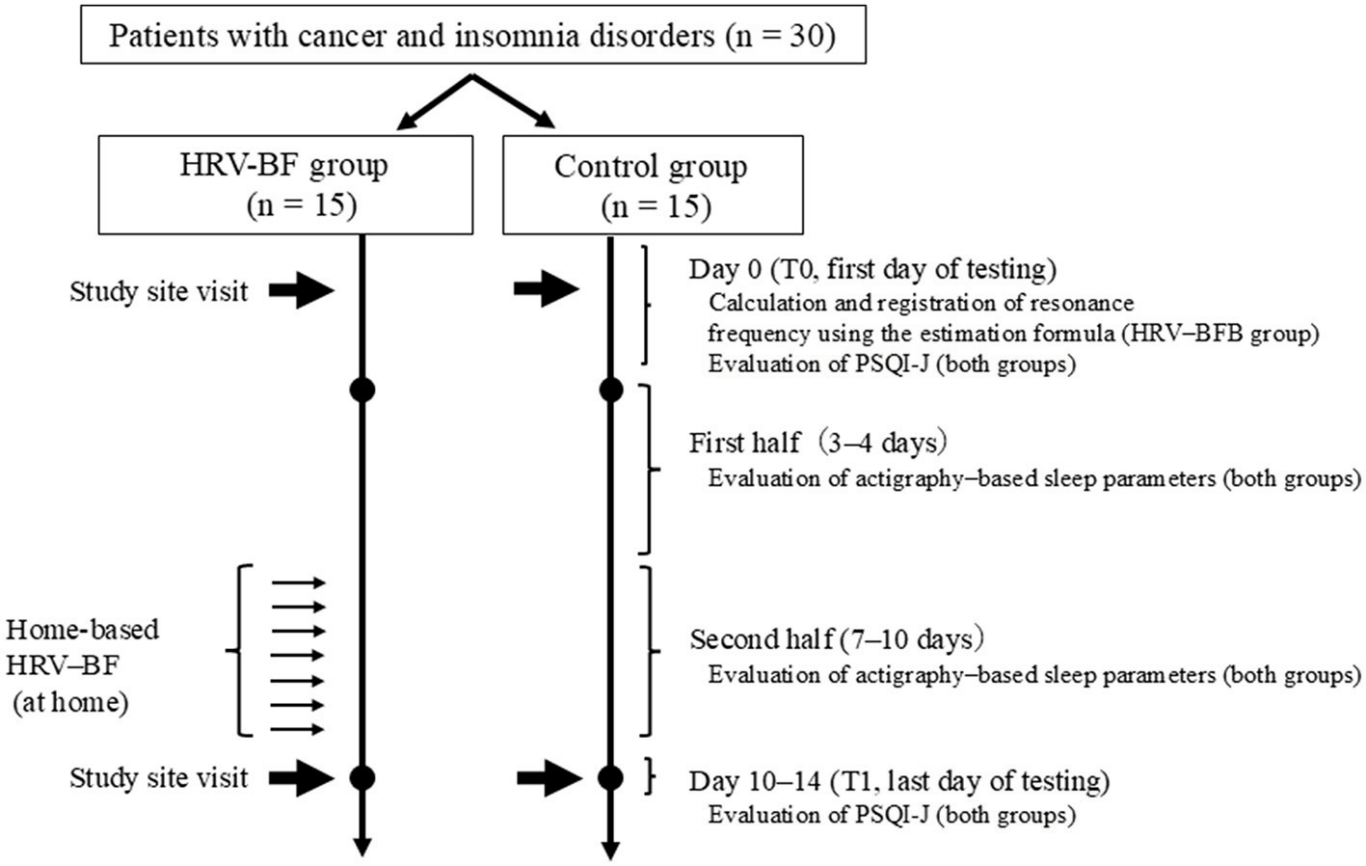
Home-based heart rate variability-biofeedback (HRV-BFB) device. The device includes a breath pacer set to match the estimated resonance frequency, guiding participants to synchronize their breathing accordingly. Indicators of synchronized breathing include a broad, large pulse wave and an elevated coefficient of variation of the RR interval (CV-RR) waveform. CV-RR, Coefficient of variation of R-R intervals.

The testing period lasted for <14 days. On day 0 (hospital visit), participants in the HRV-BFB group received portable HRV measuring devices (Panasonic AVC Networks Company, Kadoma City, Osaka, Japan; Figure 2), with their estimated resonance frequency pre-registered (T0, first day of testing). Both groups received an ActiGraph GT3X+ (ActiGraph, Pensacola, FL, USA) with usage instructions for sleep assessment (T0). This study was conducted using the same protocol as the previous study (Hasuo et al., 2023). For the second half (7–10 days) of the study period, the HRV-BFB group practiced home-based HRV-BFB sessions using the provided HRV devices for <30 min (minimum of 5 min) before bedtime. The optimal session duration has not been established; most reports describe 20-min sessions, although durations as short as 5 min and as long as 30 min have also been reported (Lalanza et al., 2023; Hasuo et al., 2023). The breath pacer in the home-based HRV-BFB device was set to match the estimated resonance frequency, and participants were instructed to synchronize their breathing accordingly. Throughout the study period (first and second half), objective sleep assessments were conducted using actigraphy. Subjective sleep assessment was conducted at baseline (T0) and on days 10–14 (T1, the last day of testing) using the self-report questionnaire, the PSQI-J (Doi et al., 2000). Sleeping medications that were used regularly or as abortives were used in conventional care at the discretion of both groups based on their sleep status. Devices were collected on T1 at the outpatient clinic.

The primary endpoint of the study was the change in sleep efficiency, assessed objectively as the ratio of total sleep duration to total sleep duration at night. Secondary endpoints included (1) changes in sleep latency, total sleep duration, wake time after sleep onset, and number of awakenings (assessed objectively); (2) changes in sleep quality, sleep latency, sleep duration, habitual sleep efficiency, sleep difficulty, hypnotic use, and daytime dysfunction (assessed subjectively); (3) sleep medication adherence (defined as use of psychotropic drugs for 3 or more days per week or at least 1 day every 2 days); (4) implementation and completion rates in the HRV-BFB group; and (5) adverse events.

Given that our data did not meet the normality assumptions required for parametric tests, analyses employed the Wilcoxon test for intragroup comparisons (T0 vs. T1) and between-group comparisons (T1–T0 difference; HRV-BFB group vs. control group) for objective sleep assessment and the sleep medication adherence (two-sided 10% significance level), with complete case analysis as the main outcome. Statistical analyses were performed using R version 4.3.2 (R Foundation for Statistical Computing, Vienna, Austria), and statistical significance was defined at *p* < 0.05, without primary analysis.

## Results

A total of 30 patients were initially enrolled from August 2022 to April 2023. However, two patients in the control group were excluded due to actigraphy failure. Patient demographics and clinical characteristics of both groups at baseline are presented in Table 1. Among survivors, two patients in the HRV-BFB group were stage I and two were stage II, whereas in the control group, three were stage I and two were stage II. All patients with incurable cancer were stage IV. Intra- and intergroup comparisons of changes in sleep parameters (T0 to T1) are presented in Table 2. For the primary endpoint, the HRV-BFB group showed significant improvement in sleep efficiency compared to the control group when assessed objectively (*p* < 0.001). Similarly, when assessed subjectively, a significant improvement was also observed (*p* = 0.009) (Table 2). On the other hand, there was little change in sleep latency and total sleep duration before and after the intervention in the HRV-BFB group, and no significant difference was observed between the groups. Regarding sleep medication adherence, 88.2% [95% confidence intervals (CIs), 73.6–95.8] in the HRV-BFB group and 90.3% (95% CIs, 74.3–97.4) in the controlled group utilized sleep medication during the first half of the study period (3–4 days). By the second half (7–10 days), sleep medication adherence dropped to 51.5% (95% CIs, 41.8–61.1) in the HRV-BFB group, whereas that increased to 92.5% (95% CI, 84.3–96.8) in the control group (between-group difference in change, *p* < 0.001). Home-based HRV-BFB sessions in the HRV-BFB group showed an implementation rate of 87.1% (95% CIs, 74.8–99.4), with an average of 8.5 ± 2.2 days for all sessions (completion rate, 100%). No adverse events were observed in either group.

**Table 1:** Comparison of baseline demographics and clinical characteristics between the HRV-BFB and control groups.

**Variable**	**HRV-BFB group**		**Control group**	
	***n*** = **15**		***n*** = **15**	
Age (year), median (min–max)	67	(44–78)	58	(49–75)
Sex (female), *n* (%)	8	(53.3)	8	(53.3)
Hight (cm), median (min–max)	163.6	(149.1–171.2)	166.2	(150.4–182.8)
**Cancer patients**				
Survivor, *n* (%)	4	(26.7)	5	(33.3)
Patients with incurable cancer, *n* (%)	11	(73.3)	10	(66.7)
Chemotherapy, *n*	9		5	
Best supportive care (palliative care), *n*	1		1	
Immuno-oncology therapy, *n*	1		0	
Chemo-radiotherapy, *n*	0		1	
Chemotherapy and palliative care, *n*	0		1	
Radiation therapy, *n*	0		2	
**Performance status**, ***n*** **(%)**				
0	5	(33.3)	3	(20.0)
1	4	(26.7)	11	(73.3)
2	4	(26.7)	0	(0.0)
3	2	(13.3)	1	(6.7)
4	0	(0.0)	0	(0.0)
Psychotropic drugs use, *n* (%)	11	(73.3)	7	(46.7)
Benzodiazepine hypnotics, *n*	4		2	
Non-benzodiazepine hypnotics, *n*	3		2	
Orexin receptor antagonists, *n*	4		3	

HRV, heart rate variability; BF, biofeedback; SD, standard deviation; IQR, interquartile range.

**Table 2:** Comparison actigraphy-based sleep parameters between the HRV-BFB and control groups.

**Sleep parameter**	**HRV-BFB group**				**Control group**						***p^b^***
	***n*** = **15**					***n*** = **13**					
	**T0 (first day of testing)**		**T1 (last day of testing)**		*P^a^*	**T0 (first day of testing)**		**T1 (last day of testing)**		*p^a^*	
	**Median**	**(Min-max)**	**Median**	**(Min-max)**		**Median**	**(Min-max)**	**Median**	**(Min-max)**		
**Actigraphy sleep parameters**											
Sleep latency, minutes	6.0	(3.0–56.7)	5.7	(2.8–8.3)	0.724	4.5	(3.3–6.3)	5.0	(1.7–6.2)	0.858	0.928
Total sleep duration, minutes	382.2	(274.2–434.3)	381.4	(44.3–547.9)	0.838	344.3	(289.3–658.8)	372.5	(251.0–550.3)	0.683	0.316
Sleep efficiency, %	82.8	(62.4–96.4)	88.8	(73.2–97.2)	0.081	85.6	(73.8–98.0)	82.2	(68.1–95.3)	0.264	<0.001
Sleep efficiency, %^&^ and mean (SD)	82.0	9.1	87.8	7.2		86.7	6.4	83.6	7.8		<0.001
Wake time after sleep onset, minutes	81.3	(13.0–222.8)	47.7	(7.0–160.8)	0.187	53.1	(6.6–102.0)	78.3	(16.0–108.6)	0.125	<0.001
Number of awakenings	21.7	(1.5–40.7)	14.7	(1.5–29.3)	0.115	15.5	(3.2–23.1)	16.4	(4.0–31.3)	0.243	<0.001
**PSQI-J score**											
Total score	12	(7–17)	7	(2–13)	<0.001	10	(5–17)	11	(6–20)	0.119	0.001
Sleep quality	2	(1–3)	1	(0–2)	<0.001	2	(1–3)	2	(1–3)	0.499	0.002
Sleep latency	2	(0–3)	1	(0–3)	0.173	1	(0–3)	1	(0–3)	0.104	0.265
Sleep duration	2	(1–3)	2	(1–3)	0.014	2	(0–3)	2	(0–3)	0.582	0.176
Habitual sleep efficiency	2	(0–3)	0	(0–3)	0.002	1	(0–3)	1	(0–3)	0.217	0.009
Sleep difficulty	1	(0–3)	1	(0–3)	0.238	1	(0–3)	1	(0–3)	0.389	0.218
Hypnotic use	3	(0–3)	1	(0–3)	0.001	2	(0–3)	2	(0–3)	0.334	0.040
Daytime dysfunction	1	(0–3)	0	(0–2)	0.006	2	(0–3)	2	(0–3)	0.271	0.005

HRV, heart rate variability; BF, biofeedback; SD, standard deviation.

a Wilcoxon test for intra-group comparisons between T0 and T1.

b Wilcoxon test for T1–T0 differences between the HRV-FB and control groups.

& Primar.

## Discussion

To the best of our knowledge, this is the first report on coping skills for patients with cancer and insomnia using a complete home-based HRV-BFB program with an estimation formula for resonance frequency. Our results confirmed two key findings.

First, despite being completely home-based, the HRV-BFB group achieved significant sleep efficiency improvement within a short period of 8.5 consecutive days, as indicated by both objective and subjective sleep data. Recent research has also reported that over a short period of 15 days with eight sessions, HRV-BFB improved sleep efficiency more than electroencephalographic biofeedback (Li et al., 2024). Zeichner et al. (2017) reported that the evaluation of cognitive behavioral therapy for insomnia and mindfulness-based resilience training as a coping skill among patients with cancer and insomnia was generally conducted after long-term interventions (6–12 weekly sessions). The faster efficacy observed in the present study (8.5 days) may be ascribed to the high implementation and completion rates of home-based HRV-BFB. Studies suggest that positive patient perceptions of HRV-BFB comfort can improve treatment expectations (Hasuo et al., 2018). Such perceptions, along with rapid efficacy, may have contributed to reduced sleep medication adherence and increased self-efficacy (Riemann and Perlis, 2009). Additionally, from the perspective of early palliative care, home-based coping skills may further enhance self-efficacy (Yoong et al., 2013).

Second, sleep efficiency is considered poor when it falls below 85% (Spielman et al., 1987). However, after the HRV-BFB group intervention, it was only slightly elevated at 87.8%. This could be ascribed to the reduction in sleep medication use during the trial period in the HRV-BFB group, which may have affected sleep efficiency, as well as the characteristics of their insomnia in this study, such as awakenings after sleep onset despite good sleep latency and total sleep time (Table 1).

The limitations of our study include (1) potential bias related to patient affiliation: the trial included only cancer survivors and patients with incurable cancer who presented to the palliative care department, so the results may not be generalizable to all cancer patients; (2) the inclusion of both survivors and patients with incurable cancer, who may have differing sleep characteristics and treatment responses; and (3) measurement variability of the HRV-BFB device, which may have affected intervention efficacy and outcome assessments. Additionally, as this study was open-label, participants' expectations may have influenced adherence and reported outcomes; (4) the study's short duration and small sample size may limit the generalizability of the results, as long-term effects and variations in a large population remain unclear.

## Conclusion

Complete home-based HRV-BFB using an estimation formula for resonance frequency may contribute to the management of patients with cancer and insomnia disorders, potentially improving sleep efficiency and reducing sleep medication reliance within a short period. Integrating HRV-BFB into standard care pathways, particularly in palliative care, could offer a practical, non-pharmacological option for improving sleep quality while minimizing hospital visits.

## Data Availability

The raw data supporting the conclusions of this article will be made available by the authors, without undue reservation.
